# Failing Homeostasis of Quadriceps Muscle Energy- and pH Balance During Bicycling in a Young Patient With a Fontan Circulation

**DOI:** 10.3389/fcvm.2019.00121

**Published:** 2019-08-21

**Authors:** Meindina G. Haarman, Johannes D. L. Vos, Rolf M. F. Berger, Tineke P. Willems, Jeroen A. L. Jeneson

**Affiliations:** ^1^Center for Congenital Heart Diseases, Department of Pediatric Cardiology, Beatrix Children's Hospital, University Medical Center Groningen, University of Groningen, Groningen, Netherlands; ^2^Department of Radiology, University Medical Center Groningen, University of Groningen, Groningen, Netherlands; ^3^Division of Neurosciences, Neuroimaging Center, University Medical Center Groningen, University of Groningen, Groningen, Netherlands

**Keywords:** congenital heart disease, univentricular cardiac disease, exercise, phosphorus-31 magnetic resonance spectroscopy, metabolism

## Abstract

**Aims:** Patients with a congenital heart condition palliated with a Fontan circulation generally present with decreased exercise capacity due to impaired cardiopulmonary function. Recently, a study in patients with a Fontan circulation reported evidence for abnormal vascular endothelial function in leg muscle. We investigated if abnormal skeletal muscle hemodynamics during exercise play a role in the limited exercise tolerance of Fontan patients. If so, abnormalities in intramuscular energy metabolism would be expected both during exercise as well as during post-exercise metabolic recovery.

**Methods:** In a young patient with a Fontan circulation and his healthy twin brother we studied the *in vivo* dynamics of energy- and pH-balance in quadriceps muscle during and after a maximal in-magnet bicycling exercise challenge using 31-phosphorus magnetic resonance spectroscopy. An unrelated age-matched boy was also included as independent control.

**Results:** Quadriceps phosphocreatine (PCr) depletion during progressive exercise was more extensive in the Fontan patient than in both controls (95% vs. 80%, respectively). Importantly, it failed to reach an intermittent plateau phase observed in both controls. Quadriceps pH during exercise in the Fontan patient fell 0.3 units at low to moderate workloads, dropping to pH 6.6 at exhaustion. In both controls quadriceps acidification during exercise was absent but for the maximal workload in the twin brother (pH 6.8). Post-exercise, the rate of metabolic recovery in the Fontan patient and both controls was identical (time constant of PCr recovery 32 ± 4, 31 ± 2, and 28 ± 4 s, respectively).

**Conclusion:** Homeostasis of quadriceps energy- and pH-balance during a maximal exercise test failed in the Fontan patient in comparison to his healthy twin brother and an age-matched independent control. Post-exercise metabolic recovery was normal which does not support the contribution of significant endothelial dysfunction affecting adequate delivery of oxidative substrates to the muscle to the lower exercise capacity in this particular Fontan patient. These results suggest that mitochondrial ATP synthetic capacity of the quadriceps muscle was intact but cardiac output to the leg muscles during exercise was insufficient to meet the muscular demand for oxygen. Therefore, improving cardiac output remains the main therapeutic target to improve exercise capacity in patients with a Fontan circulation.

## Introduction

Patients with a univentricular heart are commonly palliated with a Fontan circulation, where all systemic venous blood does not enter the heart but is diverted directly into the pulmonary arteries, without the interposition of a ventricle (1, 2). As a consequence, the single ventricular heart provides the systemic circulation in these individuals. Not surprisingly, these patients generally present with decreased exercise capacity (3–7). Classic work in exercise physiology has shown that cardiac reserve of the healthy human heart is insufficient to support adequate blood supply to the legs during maximal exercise (8, 9).

Cardiac output is, however, not the sole determinant of exercise capacity. Healthy vascular as well as skeletal muscle function also play a role (10–12). Recently, a study in patients with a Fontan circulation reported evidence for abnormal vascular endothelial function in leg muscle. On basis of this finding the authors hypothesized that decreased exercise capacity in Fontan patients may in part be caused by abnormal skeletal muscle hemodynamics (13).

Here, this matter was further investigated. In a young patient with a Fontan circulation and his healthy twin brother we studied the *in vivo* dynamics of energy- and pH-balance in quadriceps muscle during and after a maximal bicycling exercise challenge using 31-phosphorus magnetic resonance spectroscopy (31P-MRS). An unrelated sex- and age-matched boy was also studied as independent control. The aim was to investigate if abnormal skeletal muscle hemodynamics during exercise play a role in the limited exercise tolerance of Fontan patients. If so, abnormalities in intramuscular energy metabolism would be expected both during exercise as well as during post-exercise metabolic recovery.

## Materials and Methods

### Ethics Statement

This study was conducted in accordance with the Declaration of Helsinki and was approved by the institutional ethics committee (University Medical Center Groningen). Informed consent for participation and publication was obtained from all study participants and/or their legally authorized representative(s).

### Case Presentation

In this pilot study a young patient with a Fontan circulation, his healthy twin brother, and an unrelated sex- and age-matched control were included.

The patient, one of monozygotic twins, was diagnosed at birth with hypoplastic left heart syndrome due to mitral and aortic valve atresia for which he underwent a Norwood I procedure, followed by a bidirectional Glenn within the first year of life and subsequent completion into a Fontan circulation (with a fenestrated lateral tunnel) at the age of 4.5 years. Cardiac and developmental follow-up was uncomplicated and he leads an active lifestyle including weekly swimming classes, gymnastics, and biking, although his exercise tolerance is limited. Currently at the age of 11 years, he presented at the outpatient clinic.

### Measurements

Patients with a Fontan circulation are followed with a standard follow-up protocol. This includes a 2-yearly echocardiography, cardiac magnetic resonance (CMR) imaging, pulmonary function test, and a cardiopulmonary exercise test (CPET). Also, information on height, weight, heart rate, blood pressure, and transcutaneous oxygen saturation at rest are reported.

### CPET

CPET was performed on a stationary cycle ergometer with a ramp protocol with an increase of 20 W per minute. Arterial oxygen saturation was continuously monitored by transcutaneous pulse oximetry placed on the forehead. Oxygen uptake was measured using breath-by-breath analysis. The respiratory exchange ratio was calculated as the ratio between oxygen uptake (VO2) and carbon dioxide (VCO2) production at peak exercise. When an RER of >1.01 was reached, the performance was classified as adequate (14).

### 31P-MRS

Six weeks after the CPET, a second bicycling exercise test inside an MRI scanner was performed. Workload increments were derived from the results of the first CPET to ensure maximal exercise intensity was achieved within approximately 10 min. Dynamic *in vivo* 31P-MRS recordings of intramuscular energy metabolism and muscle pH were obtained from the vastus lateralis muscle at rest, during progressive exercise and subsequent metabolic recovery, respectively (15). Intramuscular phosphocreatine (PCr) concentration, a measure of muscular energy reserve, and pH were determined from the 31P-MRS recordings as described previously (15). The rate of post-exercise metabolic recovery, a measure of mitochondrial ATP synthetic function (16), was determined by non-linear curve-fitting of mono-exponential functions to the PCr and Pi time-course data weighted by SD of individual data points yielding estimates of the time constants tau_PCr and tau_Pi, respectively, as described previously (15).

The two healthy controls likewise performed the intra-MRI exercise test to obtain control data sets. Workload increments for these subjects were based on reference workload values for CPET (17).

## Results

The patient with a Fontan circulation reported no complaints of syncope, dizziness, palpitations, or chest pain. He swims once a week for 45 minutes. During gymnastics at school he sometimes needs to take a break. Besides methylphenidate, he did not use any medication. Anthropometric measurements were height: 157.8 cm (Z-score+1.00); weight: 40 kg (Z-score +0.7). Physical examination revealed that he was in good clinical condition. Blood pressures measured were 122/52 mmHg and 122/58 mmHg on his right arm and right leg, respectively. He had a normal respiratory rate and his oxygen saturation was 87%. Further physical examination showed no abnormalities besides a grade 2 systolic ejection murmur 2nd−4th left intercostal space and a palpable liver of 1 cm.

Echocardiography showed a moderate to normal systolic function of the systemic right ventricle, unobstructed cavopulmonary anastomoses, only mild atrioventricular valve insufficiency, and an open fenestration with a right to left shunt with an estimated mean pressure gradient of 6 mmHg.

During a maximal exercise test, confirmed by a RER of 1.1, oxygen saturation dropped from 88% at rest to 77% at maximal workload without any subsequent drop in O2 pulse. His maximal workload (108 Watt, 74% of predicted), maximal oxygen uptake adjusted for bodyweight (36.6 ml/kg/min, 76% of predicted), O2 pulse (77%), and maximal heart rate (164 bpm, 88% of predicted) were decreased compared to reference values (17). Patient's breathing reserve was 30% at 41 breaths/min. The VE/VCO2 slope was 40.4 and the anaerobic threshold was located at 68% of VO2 max. ECG monitoring showed a nodal cardiac rhythm at rest, rapidly converting into sinus rhythm during exercise.

### 31P-MRS Results

**Figure 1:**
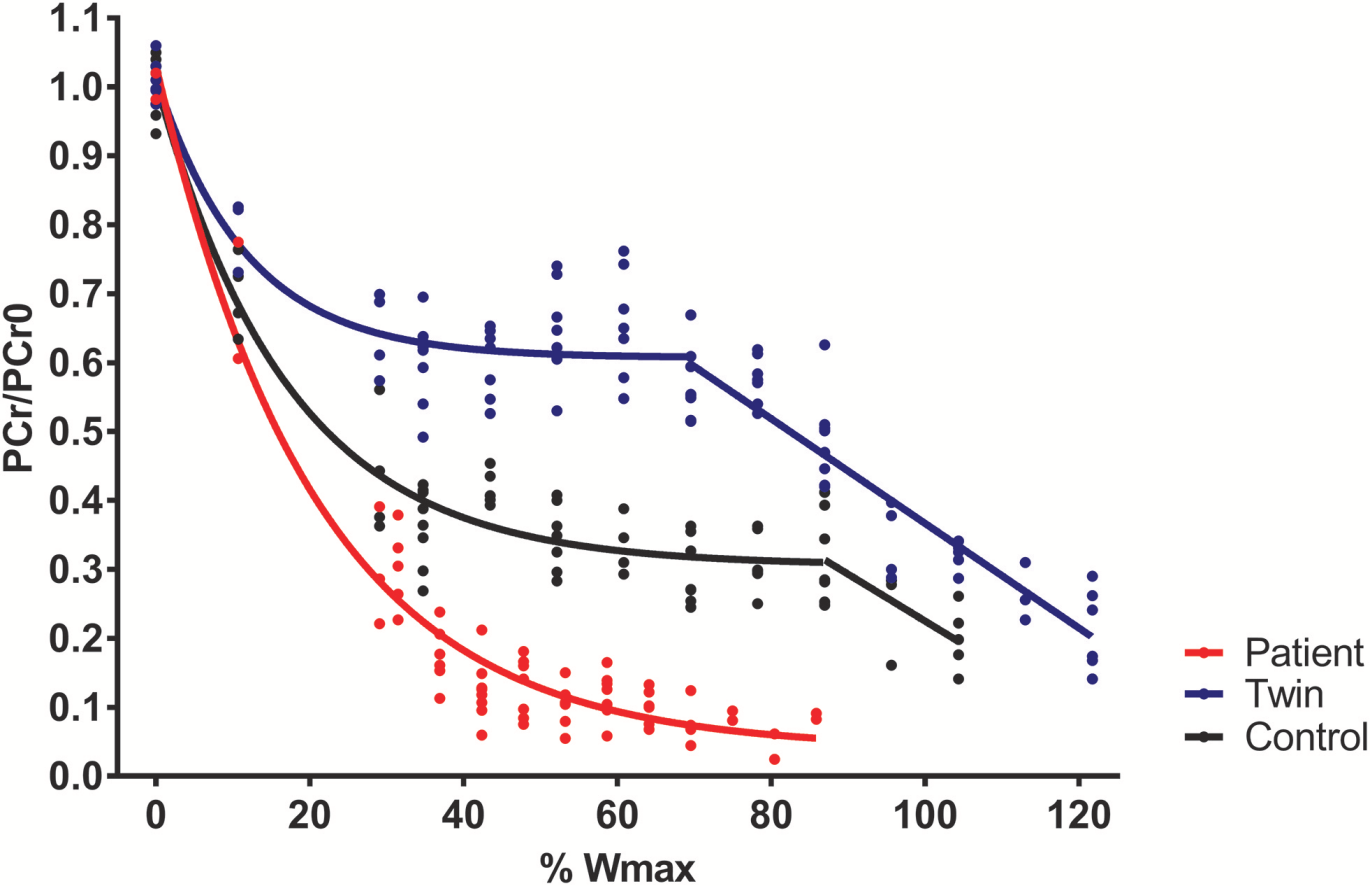
Quadriceps phosphocreatine (PCr) content (scaled to resting content) during incremental exercise recorded in a young patient with a Fontan circulation (red trace), his healthy twin brother (blue trace), and a second age- and sex-matched healthy control (black trace). Quadriceps PCr content was determined from ^31^P-magnetic resonance spectra as described elsewhere (15).

**Figure 2:**
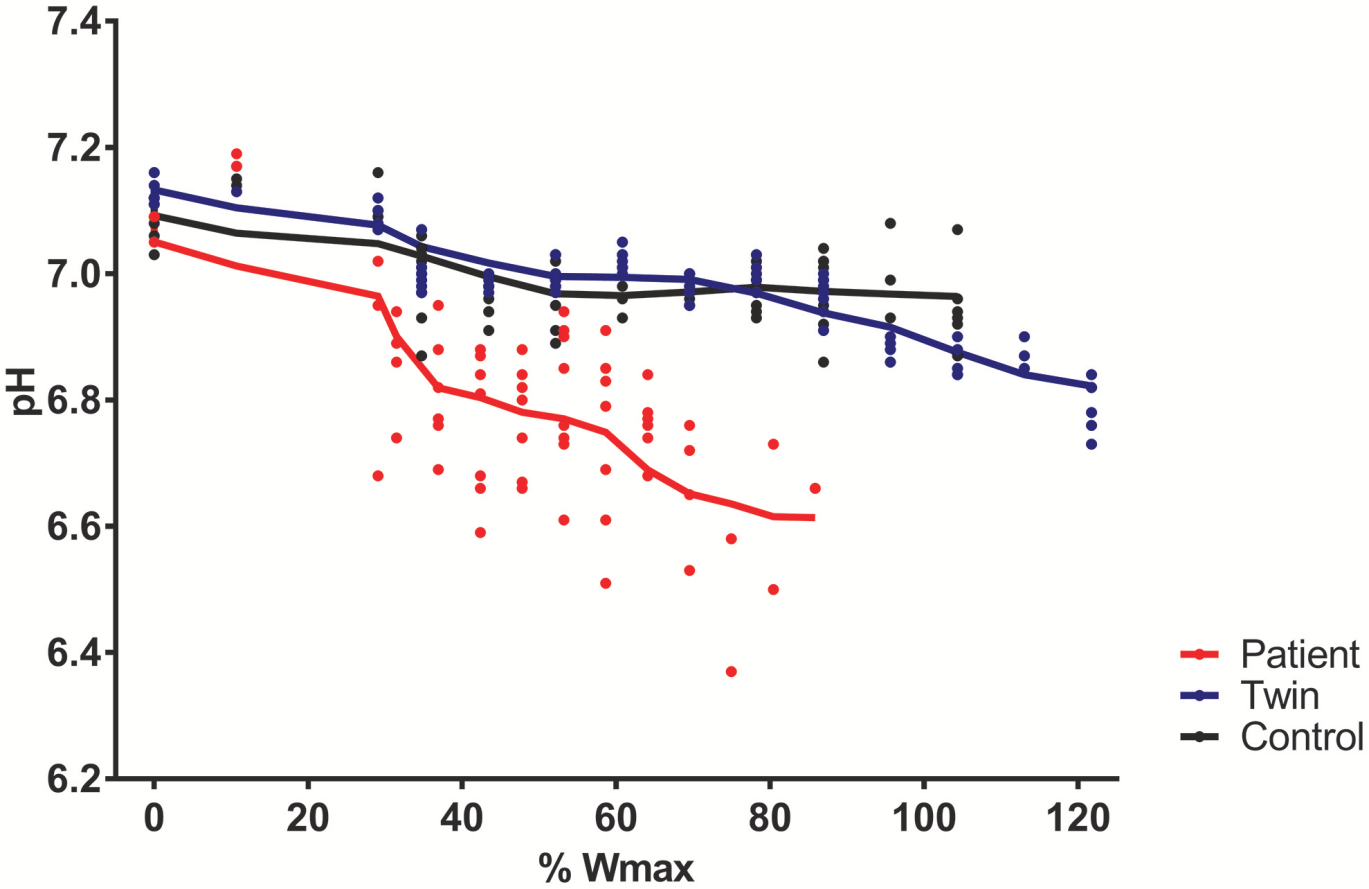
Quadriceps pH during incremental exercise recorded in a young patient with a Fontan circulation (red trace), his healthy twin brother (blue trace), and a second age- and sex-matched healthy control (black trace). Quadriceps pH was determined from ^31^P-magnetic resonance spectra as described elsewhere (15).

Total exercise time of the bicycling exercise test inside the MRI scanner of the patient, twin brother and second healthy control was 664, 632, and 608 seconds, respectively. PCr depletion was 95% in the Fontan patient vs. 80% in both healthy boys (Figure 1). End-exercise quadriceps pH was 6.6 in the Fontan patient vs. 6.8 and 6.9 in both healthy boys (Figure 2). In addition to these quantitative differences, we observed significant qualitative differences between the time-course of muscle PCr and pH in the patient and controls. Specifically, in both healthy boys, muscle PCr level attained a steady state value after an initial drop at the onset of exercise, followed by a monotonous progressive depletion at workloads above 70% (independent control) and 90% (healthy twin brother) of predicted Wmax (Figure 1). Strikingly, in the Fontan patient no such intermittent homeostatic plateau phase was observed (Figure 1). Similarly, homeostasis of quadriceps pH in both healthy boys was robust over almost the entire range of workloads, whereas in the patient progressive muscle acidification was manifest already at early stages of the ramp exercise protocol (Figure 2). Post-exercise, the rates of metabolic recovery in the Fontan patient, his twin brother and the healthy control were identical (tau_PCr recovery 32 ± 4, 31 ± 2, and 28 ± 4 seconds, respectively; tau-Pi 24 ± 4, 32 ± 2, and 30 ± 6 seconds, respectively; Supplementary Figure 1 showing the metabolic recovery in the Fontan patient). These rates are in good agreement with earlier findings in human quadriceps muscle (18, 19).

## Discussion

We have obtained *in vivo* evidence that exercise intolerance in a Fontan patient presenting with cyanosis and chronotropic incompetence, is associated with failing homeostasis of quadriceps muscle energy balance and pH during exercise. Post-exercise metabolic recovery was completely normal. These findings impact the debate on the pathophysiological basis of exercise intolerance in patients with a univentricular heart palliated with a Fontan circulation. Firstly, we found no evidence for any endothelial dysfunction in the vascular bed of the leg muscles in this particular patient. Post-exercise metabolic recovery of resting PCr and Pi levels in the quadriceps muscle of the patient followed first-, not zero-, order kinetics. Moreover, the rate of recovery was identical to the rates measured in his healthy twin brother and a second, unrelated control. The latter finding also indicates that mitochondrial ATP-synthetic function in leg muscle of the patient was intact (16).

The presence of skeletal muscle hemodynamic abnormalities in response to exercise in Fontan patients was reported by Inai et al. (13). Their near-infrared spectroscopy (NIRS) observations in 50 patients palliated with a Fontan circulation showed that post-exercise recovery of muscle oxygenation in an unspecified Fontan patient was clearly dampened both in amplitude as well as rate [Figure 2 in (13)]. It has previously been shown that post-exercise metabolic recovery in muscle studied using NIRS typically correlates well with direct measurement of intramuscular metabolic recovery using 31P-MRS (20). Therefore, the fact that we failed to find any abnormalities in post-exercise metabolic recovery in our patient using 31P-MRS rules out that vascular dysfunction in skeletal muscle of single ventricle Fontan patients is a generic feature contributing to exercise limitations in this condition.

Our results of failing homeostasis of energy- and pH-balance in quadriceps muscle during exercise in the patient despite intact mitochondrial oxidative capacity suggest that cardiac output to the leg muscles during exercise was insufficient to meet the muscular demand for oxygen. Improving cardiac output therefore remains the main therapeutic target to improve exercise capacity in Fontan patients. The challenge will be to achieve this objective in a manner that is safe for the patient. The VO2 max depends on the function of the heart, the lungs and the muscles (17). Our results question the contribution of impaired mitochondrial oxidative capacity of the leg muscles in this particular Fontan patient. Although moderate-to-vigorous aerobic and resistance exercise training in Fontan patients has shown to improve venous return via an augmented peripheral muscle pump and to improve exercise capacity, the mechanism via which this is reached seems not to be an increase in mitochondrial oxidative capacity of the leg muscles (21).

Our results are based on a small sample size and therefore any definite conclusions cannot be drawn. Future studies should include more patients with a Fontan circulation. Also, using baseline CPET values in healthy controls would be worth considering. Future studies of this subject should preferably use additional methods, including dynamic MRI methodology, to investigate the presence of endothelial dysfunction in patients with a Fontan circulation (22, 23).

An alternative strategy could be to harness the metabolic power of dietary ketones to boost cardiac performance during exercise in Fontan patients. Indeed, ketone body suppletion in rodents was found to increase cardiac hydraulic work capacity by some 25% (24, 25). A recent study in athletes has found that ingestion of a synthetic ketone ester prior to physical exercise improved performance (26). Evidence suggests that the heart will switch almost completely to ketone oxidation when this oxidative substrate is available in the bloodstream (27, 28). In humans, mild nutritional ketosis may be safely achieved either by ingestion of ketone ester (26, 29) or medium-chain triglycerides [MCT; (30)]. As such, it may be interesting to study if mild nutritional ketosis during exercise may be beneficial in Fontan patients.

## Data Availability

The datasets generated for this study are available on request to the corresponding author.

## Ethics Statement

This study was conducted in accordance with the Declaration of Helsinki and was approved by the institutional ethics committee (University Medical Center Groningen). Informed consent for participation and publication was obtained from all study participants and/or their legally authorized representative(s).

## Author Contributions

MH, JV, JJ, and RB: substantial contributions to conception and design, acquisition of data, analysis, interpretation of data, drafting the article, and revising it critically for important intellectual content. RB and TW: revising the article critically for important intellectual content. All authors: final approval of the version to be published.

### Conflict of Interest Statement

The University Medical Center Groningen has contracts with Actelion and Lilly for consultancy-activities of RB, outside the submitted work. The remaining authors declare that the research was conducted in the absence of any commercial or financial relationships that could be construed as a potential conflict of interest.

## Supplementary Material

Supplementary Figure 1 Quadriceps phosphocreatine (PCr) recovery after incremental exercise in a young patient with a Fontan circulation. Red line, a mono-exponential fit of the data (16); dotted blue lines, 95% confidence interval of the mono-exponential fit of the data (*R*^2^ = 0.84; tau_PCr recovery = 32 ± 4 seconds). Quadriceps PCr content was determined from ^31^P-magnetic resonance spectra as described elsewhere (15).
