# Supplementary figures and images for: Failing Homeostasis of Quadriceps Muscle Energy- and pH Balance During Bicycling in a Young Patient With a Fontan Circulation
Source: Front Cardiovasc Med. 2019 Aug 21;6:121. doi: 10.3389/fcvm.2019.00121 (PMC6712941; doi:10.3389/fcvm.2019.00121)

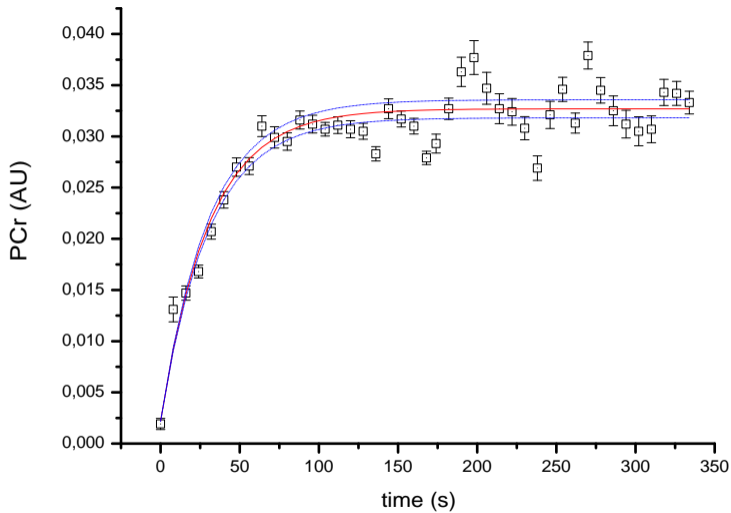

Supplement: Supplementary Figure 1 — Quadriceps phosphocreatine (PCr) recovery after incremental exercise in a young patient with a Fontan circulation. Red line, a mono-exponential fit of the data (16); dotted blue lines, 95% confidence interval of the mono-exponential fit of the data (R2 = 0.84; tau_PCr recovery = 32 ± 4 seconds). Quadriceps PCr content was determined from 31P-magnetic resonance spectra as described elsewhere (15). [file Image_1.pdf]
